# Systemic treatment of xenografts with vaccinia virus GLV-1h68 reveals the immunologic facet of oncolytic therapy

**DOI:** 10.1186/1471-2164-10-301

**Published:** 2009-07-07

**Authors:** Andrea Worschech, Nanhai Chen, Yong A Yu, Qian Zhang, Zoltan Pos, Stephanie Weibel, Viktoria Raab, Marianna Sabatino, Alessandro Monaco, Hui Liu, Vladia Monsurró, R Mark Buller, David F Stroncek, Ena Wang, Aladar A Szalay, Francesco M Marincola

**Affiliations:** 1 Genelux Corporation, San Diego Science Center, San Diego, California, USA; 2 Virchow Center for Experimental Biomedicine and Institute for Biochemistry, University of Würzburg, Am Hubland, Würzburg, Germany; 3 Infectious Disease and Immunogenetics Section (IDIS), Department of Transfusion Medicine, Clinical Center, National Institutes of Health, Bethesda, Maryland, USA; 4 Department of Molecular Microbiology and Immunology, Saint Louis University School of Medicine, St Louis, MO, USA; 5 Cellular Processing Section, Department of Transfusion Medicine, National Institutes of Health, Bethesda, Maryland, USA; 6 Department of Pathology, Immunology Section, University of Verona Medical School, Verona, Italy

## Abstract

**Background:**

GLV-1h68 is an attenuated recombinant vaccinia virus (VACV) that selectively colonizes established human xenografts inducing their complete regression.

**Results:**

Here, we explored xenograft/VACV/host interactions *in vivo *adopting organism-specific expression arrays and tumor cell/VACV *in vitro *comparing VACV replication patterns. There were no clear-cut differences *in vitro *among responding and non-responding tumors, however, tumor rejection was associated *in vivo *with activation of interferon-stimulated genes (ISGs) and innate immune host's effector functions (IEFs) correlating with VACV colonization of the xenografts. These signatures precisely reproduce those observed in humans during immune-mediated tissue-specific destruction (TSD) that causes tumor or allograft rejection, autoimmunity or clearance of pathogens. We recently defined these common pathways in the "immunologic constant of rejection" hypothesis (ICR).

**Conclusion:**

This study provides the first prospective validation of a universal mechanism associated with TSD. Thus, xenograft infection by oncolytic VACV, beyond offering a promising therapy of established cancers, may represent a reliable pre-clinical model to test therapeutic strategies aimed at modulating the central pathways leading to TSD; this information may lead to the identification of principles that could refine the treatment of cancer and chronic infection by immune stimulation or autoimmunity and allograft rejection through immune tolerance.

## Background

In the past, we applied inductive reasoning [1] to identify immunologic signatures associated with tumor rejection, clearance of pathogen, acute allograft rejection or autoimmunity. This exercise leads to the formulation of the "immunologic constant of rejection" (ICR) hypothesis: "*immune-mediated tissue specific destruction (TSD) follows a common final pathway independent of the originating cause and the disease context*" [2]. 4 axioms were proposed at the basis of the ICR: *i*) TSD does not necessarily occur because of non-self recognition but also occurs against self or quasi-self; *ii*) the requirements for the induction of a cognate immune response differ from those necessary for the activation of an effector one; *iii*) although the prompts leading to TSD vary in distinct pathologic states, the effector immune response converges into a single mechanism; and *iv*) adaptive immunity participates as a tissue-specific trigger, but it is not always sufficient or necessary. Here, we applied deductive reasoning to test whether immunologic markers of ICR could be predictably observed in a controllable experimental model. We selected a promising pre-clinical endeavor where the systemic administration of oncolytic VACV induces xenograft regression in immune deficient mice through an, at least in part, immunologically-mediated mechanism. In addition, we tested the validity of the fourth axiom, which postulates, in concordance with others' observations, that tumor rejection does not require adaptive immunity [3].

Systemic delivery of oncolytic viruses leads to their specific localization to established tumors and to viral replication followed by oncolysis [4]. VACV, in particular, possesses strong antitumor properties [5] while its history as vaccine against smallpox proves it safe in humans. Further attenuation by disruption of non essential viral genes such as J2R (coding for thymidine kinase) [6,7] and A56R (coding for haemagglutinin) [8] increased the therapeutic potential of VACV as an oncolytic agent. In addition, the same construct could be used for tumor-specific delivery of light-emitting proteins for real-time imaging [9] or therapeutic proteins such as tumor suppressors [10], anti-angiogenesis factors [11] or immune modulators [12]. The design of a VACV construct, GLV-1h68, derived from LIVP wild-type strain by insertion of 3 expression cassettes encoding *Renilla *luciferase-*Aequorea *green fluorescent fusion protein (RUC-GFP), β-galactosidase and β-glucoronidase [9,13] lead to a highly attenuated oncolytic virus capable of targeting established human xenografts. The ability to replicate specifically within tumors and to leave non malignant tissues virus-free makes GLV-1h68 systemic administration a promising pre-clinical tool capable of safely eradicating pancreatic cancer [14], malignant pleural mesothelioma [15], breast carcinoma GI-101A xenografts [13] and anaplastic thyroid cancer [16].

Eradication of established human breast cancer GI-101A xenografts can be reproducibly induced in nude mice injected intravenously with 1 × 10^7 ^plaque forming units (PFU) of GLV-1h68. Tumor eradication occurs in 95% of treated animals within 130 days from injection and it is associated with pristine tropism of GLV-1h68 for the xenograft and lack of systemic toxicity or mortality. Because GLV-1h68 encodes a luciferase reporter, it is possible to estimate kinetically virus titers in tumor xenografts and correlate this parameter with treatment outcome [13].

Experimental observations demonstrated a tight relationship between virus replication within the tumor xenograft and response to oncolytic treatment. However, the mechanisms leading to tumor regression by oncolytic virus remain unknown [17,18]. While it is possible that a direct oncolytic activity may be responsible for tumor regression, it is also possible that tumor eradication is the result of a complex interplay among virus, cancer cells and the host [19]. Expression profiling of xenografts responding to treatment with GLV-1h68 based on a mouse-specific platform and hence representative of the host's response to the GLV-1h68-infected human xenograft suggested that their eradication is associated with the over-expression of signatures consistent with innate immune defense activation. These signatures are inclusive of interferon-stimulated genes (ISGs) such as *STAT-1 *and *IRF-7*, chemokines (*Ccl2, Ccl9, Ccl27, Cxcl9, Cxcl10, Cxcl12*), chemokine receptors (*Ccr2*), interleukins (*IL-18*) and innate immune effector functions (IEF) [13]. The participation of immune cells was supported by immunohistochemistry, which demonstrated active peri-tumoral and intra-tumoral infiltration of monocytes in treated samples [13]; however, the specificity of the association between xenograft eradication and immune activation could not be determined since non-responding xenografts were not included in the previous analysis.

We hypothesized that in this model the eradication of responding xenografts is, at least in part, mediated through innate immune mechanisms and, as a consequence, this model could provide important insights about the role played by innate immunity in mediating tissue rejection in the immune incompetent host [2]. Recent animal studies suggest that immune-mediated eradication of syngeneic tumors is independent of adaptive immune responses [3], and the involvement of cytotoxic T cells may provide primarily help for the *in situ *targeting and/or activation of innate immune effector cells [20]. Therefore, progression of events leading to xenograft rejection in a mouse model deprived of adoptive immune function may simplify the identification of the requirements for tumor rejection and, more broadly, those necessary for TSD [2].

To determine which innate responses and virus replication characteristics specifically correlated with oncolytic GLV-1h68-mediated tumor rejection, we tested human cancer cell lines of different tissue derivation for their *in vitro *permissivity to GLV-1h68 replication, their *in vivo *colonization and their susceptibility to VACV-mediated eradication. In addition, we used 3 array platforms to characterize VACV, human (tumor cells) and mouse (inflammatory cells) gene expression in GLV-1h68-infected xenografts *in vivo*. The results demonstrated that tumor rejection is associated with activation of innate immune mechanisms in the host that recapitulate faithfully the biological pathways observed in association with immune-mediated TSD in humans [2]. Thus, this model suggests that immune rejection does not depend upon adaptive immunity as long as the initiating mechanism (in this case selective viral localization at the tumor) is specific to a particular tissue. The demonstration that immune deficient mice can reject human xenograft following a pathway common to other human immune pathologies suggests that the ICR is a universal phenomenon across species and may represent a target for immune modulation in the context of various diseases.

## Results

### Variability of xenograft responses to the systemic administration of GLV-1h68

**Figure 1:**
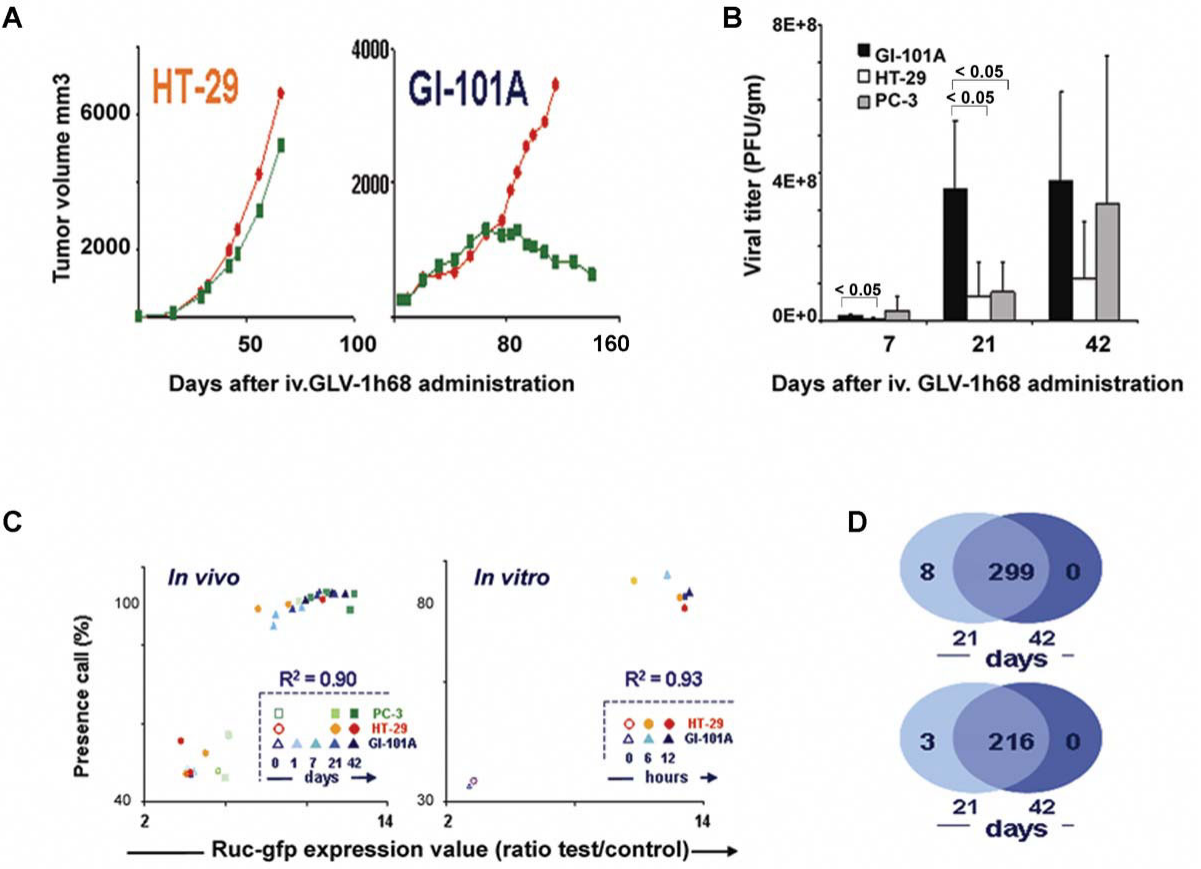
**Characterization of human xenografts and Vaccinia Virus signatures**. (**A**) Representative growth curves (n = 8 animals) for 2 xenografts from HT-29 and GI-101A cell lines; red dots represent control, green boxes the post treatment groups; for further details about the xenograft model refer to references [9,13]. (**B**) Viral titer (PFU/gram of xenograft; n = 4) comparing the permissivity of 3 xenografts derived from GI-101A, HT-29 and PC-3 cell line whose responsiveness is under characterization 7, 21 and 42 days after GLV-1h68 administration. (**C, left**) Scatter plot correlating the level of *Renilla *luciferase-*Aequorea *green fluorescent protein messenger RNA expression with the presence call of probes above the set threshold level for the VACV expression array platform. VACV gene expression in non-infected PC-3, HT-29 and GI-101A xenografts was compared to infected xenografts 1, 7 (GI-101A only), 21 and 42 days before. High presence call (> 40%) in the non-infected xenografts could be expected due to the large number of mouse and human housekeeping genes present in the array platform); R^2 ^value refers to the correlation between RUC-GFP expression and number of VACV transcripts significantly up regulated in the same experiment. (**C, right**) Scatter plot as per panel **C, left**, comparing *in vitro *VACV gene expression of GLV-1h68-infected HT-29 and GI-101A cell lines at 6 and 12 hours with controls. (**D**) A Venn diagram displays the extent of overlap among VACV-specific probes (top) and VACV genes (bottom) differentially expressed by infected GI-101A xenografts at day 21 and 42 compared with day 1 after GLV-1h68 injection.

A panel of human cancer cell lines of different histological derivation was tested for their sensitivity to the oncolytic activity of intra-venously injected GLV-1h68 [21-25]. 2 characteristic patterns were identified: some cell lines progressively continued their growth independent of therapy (i.e. HT-29), while others followed 3 growth phases: first, a slightly faster growth of infected compared to control tumors, then a period of no or minimal growth, and finally, complete regression (i.e. Gl-101A) [13] (Figure 1A). Tumor growth or regression patterns were cell line-specific, highly reproducible, and independent of number of cancer cells administered or dose of GLV-1h68 injected [13]. A therapeutic index (T.I.) was calculated to provide a single parameter descriptive of each cell line's responsiveness to VACV therapy by integrating the areas between the median growth of control and treated xenografts (eight animals in each group in all experiments described here and thereafter) (Table 1). The same cell lines were subjected to an *in vitro *assay in which their permissivity to GLV-1h68 replication was tested (**data not shown**, see Additional file 1). We observed that 3 of 3 cell lines that resisted replication during the first 24 hours following infection (MDA-MB-231, SiHa and NCI-H1299) uniformly produced xenografts partially or non-responding to VACV therapy *in vivo*. However, 8 of 10 cell lines that allowed viral replication in the first 24 hours yielded xenografts responsive *in vivo *to VACV treatment while 2 (HT-29 and 1936-MEL) yielded xenografts that did not respond. The relationship between the permissivity of a given cell line to *in vitro *replication of GLV-1h68 and the *in vivo *responsiveness of the corresponding xenograft was significant (Fisher exact test p_2_-value = 0.005) but not absolute.

**Table 1:** Therapeutic Index (T.I) of responding compared to non-responding xenografts

**Responders (R)**		T.I.	**Poor/Non-Responders (NR)**		T.I.
1858-MEL	Melanoma	90.1	MDA-MB-231	Breast Adenocarcinoma	21.6

888-MEL	Melanoma	88.0	SiHa	Cervical Squamous Cell Carcinoma	15.6

MIA PaCa-2	Pancreatic Carcinoma	80.1	1936-MEL	Melanoma	13.7

A549	Lung Carcinoma	62.8	NCI-H1299	Breast Adenocarcinoma	-2.3

OVCAR-3	Ovarian Adenocarcinoma	56.2	HT-29	Colorectal Carcinoma	-19.0

Panc-1	Pancreatic Carcinoma	50.9			

DU-145	Prostate Carcinoma	48.4			

GI-101A	Breast Carcinoma	27.9			

Of particular interest was a pair of cell lines: the colorectal carcinoma HT-29 (non responding) and the breast adenocarcinoma GI-101A (responding) cell lines [13] that displayed *in vitro *a similar degree of permissivity to VACV replication. Since the distinct behavior of the 2 cell lines could have been due to their diverse ontogeny [26], we tested a pair of autologous melanoma cell lines, 888-MEL and 1936-MEL derived from the same progenitor cell clone though established from 2 metachronous metastases [24,27]; 888-MEL was generated in 1989 during the earlier stage of disease at a time when the patient underwent a complete remission following adoptive transfer of tumor infiltrating lymphocytes; 1936-MEL was expanded 12 years later from a metastasis excised at a time when the patient was rapidly progressing and did not respond to further therapy [24]. The cell lines displayed the same degree of permissivity *in vitro *to GLV-1h68 replication, but yielded xenografts with disparate sensitivity to VACV treatment *in vivo *(Table 1). These data suggest that even in autologous systems responsiveness is related to biological characteristics of the tumors that are independent of their ontogeny, and are more likely related to evolving phenotypes during the natural history of the disease.

We then analyzed VACV replication *in vivo *in a responding (GI-101A), a non-responding (HT-29) and another presently less characterized (PC-3) line. Twenty-one days post GLV-1h68 administration; viral titers were lower in non-responding xenografts (Figure 1B). The difference was less pronounced after 42 days, suggesting that the lack of responsiveness to oncolytic therapy may be associated with delayed but not completely absent VACV replication.

### Transcriptional profiling of VACV/tumor/host interactions

To gain better insights on the mechanisms governing xenograft rejection, we compared simultaneously the transcriptional patterns of VACV, human cancer cells and mouse host cells in responding and non-responding xenografts excised at time points associated with tumor and viral growth (day 21 after injection) or at the plateau phases preceding tumor rejection (day 42). This was achieved by the adoption of organism-specific platforms.

### Transcriptional differences between xenografts responding or non-responding to systemic GLV-1h68 administration: the VACV signatures

VACV-gene expression was assessed by a custom-made VACV array platform to compare the expression of VACV transcripts *in vivo *in the responding GI-101A and the non-responding HT-29 (characterized by normal *in vitro *but delayed *in vivo *replication) xenografts. VACV transcriptional patterns correlated perfectly with viral titers. Moreover, in all cell lines there was a perfect correlation between RUC-GFP expression and overall expression of VACV genes (R^2 ^= 0.90) suggesting that this reporter gene accurately represents GLV-1h68 replication (Figure 1C). Variation in VACV gene expression was observed among cell lines or among xenografts derived from the same cell line. Furthermore, a clear dichotomy was observed in transcriptional patterns: VACV transcripts were either all up regulated or completely silent suggesting that the transition from early to late VACV gene expression occurred in rapid succession that could not be discriminated by the time points examined. Most GI-101A xenografts demonstrated early *in vivo *replication with 3 out of 4 expressing VACV genes at day 7, and 4 out of 4 at day 21 and 42. In contrast, HT-29 suffered delayed replication *in vivo *with only a proportion displaying full VACV gene expression at day 21 (2 of 4 in either case). After 42 days the expression of VACV genes was turned on only in 1 of 4 HT-29 xenografts. VACV gene expression analysis confirmed lack of differences in VACV transcriptional patterns *in vitro *between HT-29 and GI-101A with 3 out of 3 cell cultures demonstrating active viral replication in either case in the first 12 hours after infection (Figure 1C).

Subsequently, comparisons were made between infected and non-infected GI-101A and HT-29 xenografts. Even though there might have been better responder/non-responder pairs to choose from we selected HT-29 tumors among non-responding cell lines because of its similar *in vitro *permissivity to GLV-1h68 that corresponded to a different behavior *in vivo *and previous characterizations in our laboratory. A high-stringency (p_2_-value < 0.005) Student *t *test comparing the number of VACV genes differentially expressed at day 21 or 42 from infected animals with those from uninfected ones identified significant differences (multivariate permutation p-value < 0.001) only in GI-101A xenografts at day 21 and day 42 (Table 2). As previously discussed, the number of genes differentially expressed in xenografts with replicating VACV reflected completely the number of VACV-specific annotations present in the VACV-array platform demonstrating that GLV-1h68 replication is either absent or complete in xenografts at this time point. As to be expected, an almost complete overlap of VACV probes or genes expressed at day 21 and 42 was observed in the GI-101A xenografts (Figure 1D). Notably, a reverse behavior was observed in the pattern of expression of human house keeping genes represented in the VACV array platform. The expression of these genes was profoundly down-regulated in permissive cell lines suggesting a shut off of cellular metabolism in infected cells that correlated inversely with viral transcription as described by others [28]. It is noteworthy that, although HT-29 did not display significant up regulation of VACV genes using the high stringency parameters adopted here, it displayed a similar trend in gene expression with mild up-regulation of VACV genes and expression of GFP messenger RNA in some but not all xenografts (Figure 1C).

**Table 2:** Number of genes over-expressed in xenografts excised from GLV-1h68 infected animals

Experimental group	Days after VACV inj.				
**VACGLa 520445F, Affymetrix platform**		VACV only	Permutation	House keeping (human/mouse)	Permutation


(cut off p^2^-value < 0.005 (unpaired Student *t *test)		(∑ 219)	test p-value	(∑ 337)	test p-value

GI-101A	7	0	N.S.	3	N.S.

GI-101A	21	219	< 0.001	(232)	< 0.001

GI-101A	42	216	< 0.001	(237)	< 0.001

HT-29	21	0	N.S.	(3)	N.S.

HT-29	42	0	N.S.	(10)	N.S.



**37 K whole genome HUMAN array**		All genes	Permutation		
			
(cut off p^2^-value < 0.001 (unpaired Student *t *test)		(∑ 37 K)	test p-value		


GI-101A	21	136	< 0.05		

GI-101A	42	91	< 0.05		

HT-29	21	4	N.S.		

HT-29	42	10	N.S.		



**37 K whole genome Mouse array**		All genes	Permutation		
			
(cut off p^2^-value < 0.001 (unpaired Student *t *test)		(∑ 37 K)	test p-value		

GI-101A	21	105	< 0.05		

GI-101A	42	1026	< 0.001		

HT-29	21	7	N.S.		

HT-29	42	14	N.S.		

* In parenthesis, genes down-regulated in GLV-1h68-infected animals referring to human/mouse house-keeping genes in the VACV chip

### Transcriptional differences between xenografts responding or non-responding to systemic GLV-1h68 administration: the human cancer signatures

**Figure 2:**
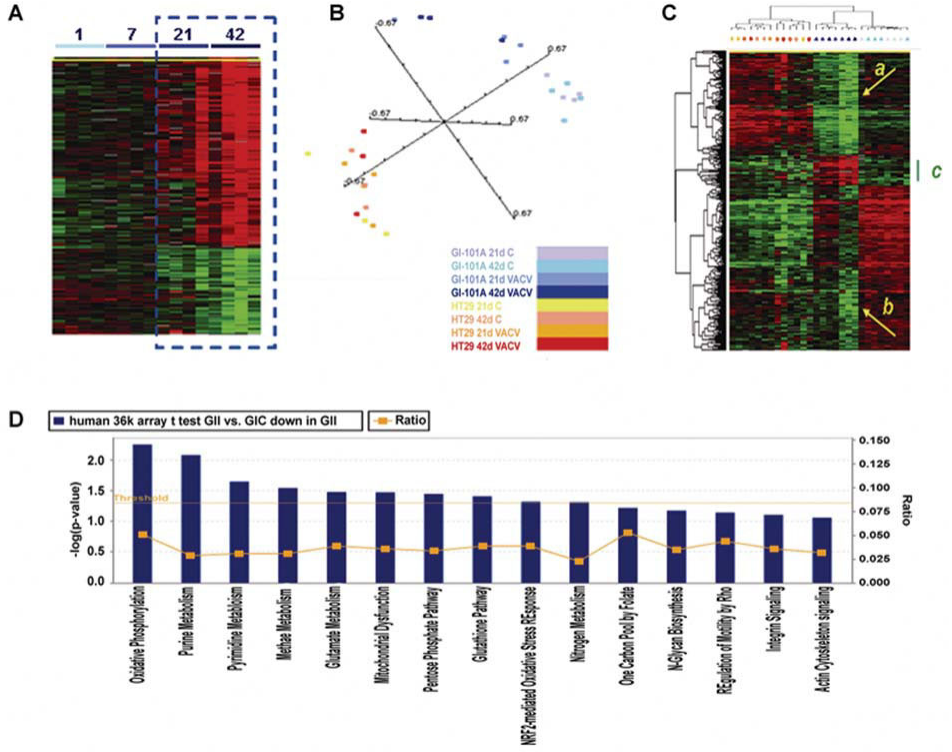
**Human Cancer signatures**. (**A**) Time course analysis of infected GI-101A xenografts (parameters for gene selection; F test p-value < 0.005, 80% presence call, ratio of > 2 and false discovery rate < 0.1). Gene distribution is shown based on 893 genes of 17,500 present in the human cDNA platform that passed the statistical criteria and presented according to Spearman rank correlation. The dashed box outlines the 2 time points most affected by VACV infection. The heat map information is presented according to the central method for normalization [30]. (**B**) Multiple dimensional scaling based on the 36 k oligo array human platform comparing HT-29 and GI-101 xenografts. (**C**) Self organizing heat map based on 841 out of 1,073 genes differentially expressed between GI-101A xenografts from infected compared to non-infected mice that passed the standard filter conditions (presence call in at least 80% and at least 3 fold ratio change). HT-29 samples are also represented as a reference, color coding of samples is as per panel (B). The green bar underlines the genes specifically expressed by Gl-101A xenografts from infected animals; the 2 yellow arrows (*a*) and (*b*) point at genes who expression was profoundly depressed in xenografts from infected animals. (**D**) Ingenuity pathway analysis showing canonical pathways significantly down-regulated in GI-101A xenografts at day 41 following GLV-1h68 injection; IPA analysis based on an unpaired, two-tailed Student *t *test comparing infected to non-infected GI-101A xenografts at day 42 (threshold p_2_-value < 0.001).

A time course analysis evaluating the *in vivo *effects of viral replication on the permissive GI-101A human xenografts was performed using a custom-made 17.5 k human cDNA array platform [29]. 4 experimental groups of 4 mice each received systemic GLV-1h68 administration 1, 7, 21 and 42 days before xenograft excision (Figure 2A[30]). The transcriptional profile of infected GI-101A tumors was altered significantly by 21 days and increasingly so at 42 days after GLV-1h68 administration. Since the time course demonstrated that even in permissive xenografts significant changes occurred only at 21 and 42 days, we limited the subsequent analysis to these time points.

To better characterize the transcriptional program of VACV-infected cancer cell lines, we compared responding (GI-101A) and non-responding (HT-29) xenografts using a 36 K whole genome, human oligo array platform at day 21 and 42. Multiple dimensional scaling based on the complete data set demonstrated that infected GI-101A xenografts (2 darker blue color) segregated completely in Euclidian space from non-infected xenografts (2 lighter blue colors) while HT-29 xenografts intermingled whether they were from infected or non-infected animals (Figure 2B).

To test overall differences between xenografts from infected and non-infected animals, we applied a Student *t *test (cutoff p_2_-value < 0.001) comparing infected to non-infected GI-101A and HT-29 xenografts. Comparison of GI-101A identified 1,073 genes differentially expressed between infected and non-infected xenografts (permutation test p value = 0). On the contrary, only 9 genes were found to be differentially expressed by HT-29 xenografts from infected compared to non-infected animals (permutation test non significant). Among genes differentially expressed in the GI-101A xenografts from GLV-1h68 infected animals, the large majority were down-regulated, particularly, in xenografts excised at day 42 suggesting that viral replication depresses cellular metabolism (Figure 2C, yellow arrows annotated with *a *and *b*) consistent with the down-regulation of house keeping genes observed in the VACV-chip. Ingenuity pathway analysis (IPA) demonstrated that, among the canonical pathways, the top 10 categories of down-regulated genes represented depressed cellular function including alterations in oxidative pathways, mitochondrial dysfunction, and disruption of purine, pyrimidine and amino acid metabolism (Figure 2D). Interestingly, a smaller cluster of genes was over-expressed in GI-101A xenografts from infected animals (green bar, Figure 2C, Table 2). Among these genes, allograft inflammatory factor-1 (AIF-1), the tissue inhibitor of metalloproteinase 2 (TIMP-2) and the IL-2 receptor common γ chain were up regulated. Moreover, a multivariate analysis (F test, p-value cutoff < 0.001) based on the oligo arrays comparing the 4 groups at day 21 and 42 (HT-29 and GI-101A in infected and non-infected mice) identified respectively 2,241 and 1,984 clones differentially expressed among the 4 groups. Analysis with the 17 k cDNA arrays similarly identified 1,467 cDNA clones representative of the 4 categories at day 42. In both platforms, most of the differences in expression pattern involved tumor cell specific genes and both platforms segregated the HT-29 xenografts from GI-101A xenografts independent of GLV-1h68 administration according to their different ontogeny; a phenomenon we have previously described [26]; however, a subgroup of genes was observed to be specific for GI-101A infected xenografts. The GLV-1h68 infection-specific signatures were enriched for up regulated genes associated with immune function with a significantly higher than expected frequency (1.88) according to GeneOntology assignment. Among the genes up-regulated in the GI-101A xenografts excised from GLV-1h68 infected mice, several were associated with activation of innate immune mechanisms including the Toll-like receptor (TLR)-2, the interferon regulatory factor (IRF)-7, signal transducer and activator of T cell (STAT)-3 and tumor necrosis factor (TNF)-α. This enrichment was not observed in oligo-based arrays suggesting that these signatures could be potentially attributed to host infiltrating immune cells whose genes could cross-hybridize to the less stringent cDNA array probes; this could occur in spite of the intensity filter adjustment for sequences with high mouse to human similarity. Sequence verification, demonstrated that only STAT-3 and IRF-7 were indeed expressed by human cells while the other genes were mouse transcripts cross hybridizing to the cDNA probes but not to the more stringent oligo-probes (see Additional file 2).

In summary, analysis of human transcripts demonstrated that differences among xenografts from infected and non-infected mice are non-existent in non-responding tumors and limited to a small set of up-regulated genes in responding tumors several of them representing over-expression of host's genes cross hybridizing to the human platform. Most transcriptional differences in responding tumors were instead due to the shut down of cellular metabolisms induced by active viral infection while little evidence of apoptotic or necrotic induction by the oncolytic process could be identified at this early time point suggesting that at day 42 cells are starting to be strongly altered in their metabolism but are still alive; this finding correlates with the presence of viable cancer cells observed by histopathological examination [13].

### Transcriptional differences between xenografts responding or non-responding to systemic GLV-1h68 administration: the mouse host's signatures

**Figure 3:**
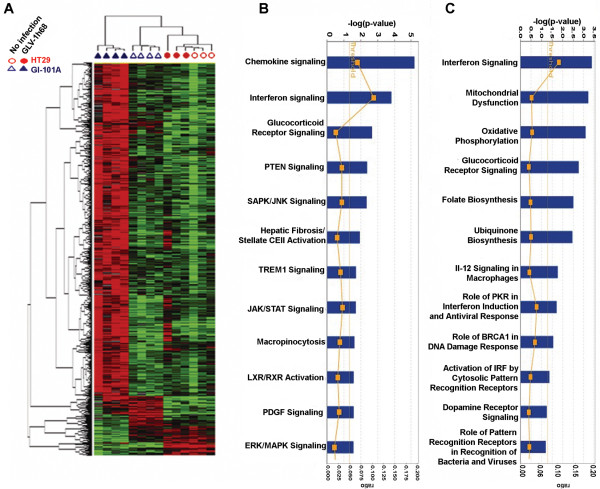
**Mouse host's signatures**. (**A**) Self-organizing heat map of mouse genes differentially expressed among the 4 experimental groups (HT-29 and GI-101A xenografts from GLV-1h68-infected or non-infected mice) according to an F test at day 42 after infection. Standard filter was applied (80% presence call, 3 fold ratio cut off) that allowed 819 out of 1,066 genes at day 21 and 1,159 out of 1,471 genes at day 42. IPA of canonical pathways over-induced in infected compared to non-infected GI-101A xenografts at day (**B**) 21 and (**C**) 42 based on an unpaired, two-tailed Student *t *test comparing infected to non-infected GI-101A xenografts (threshold p_2_-value < (A) 0.001 and (B) 0.0001).

To define the host's involvement in the early phases of the oncolytic process when tumor cells are still present and alive [13], we analyzed HT-29 and GI-101A xenografts using a custom-made, whole genome 36 K mouse array platform. In this case, all 4 GI-101A xenografts excised at day 42 from infected mice could be utilized while only 3 of 4 could be utilized for the human arrays because of degradation of human mRNA in one of the regressing xenografts. Gene expression was affected significantly only in GI-101A xenografts (Table 2). A statistical overview of gene expression modulation of GI-101A xenografts from GLV-1h68 infected grafts gave a completely opposite picture compared to the human arrays in which a predominant down-regulation of cellular metabolism was observed. In particular, most mouse genes were up regulated in xenografts excised from infected animals suggesting that, while the metabolism of cancer cell was declining (Figure 2C), the host response was enhanced (Figure 3A). An F test was performed for a global comparison of all experimental groups; at day 21, 1,066 genes demarcated the differences among the 4 experimental groups. This number increased to 1,471 by day 42 (permutation test p-value < 0.001 in either case).

At day 21, IPA reveled that the 2 canonical pathways predominantly affected in GI-101A xenografts from VACV-infected mice reflected chemokine and IFN signaling (Figure 3B). At day 42, additional canonical pathways became affected including those associated with cellular stress (Figure 3C). There was significant overlap among the 2 time points when only genes associated with immune function were compared (Table 3 and 4), while most differences between the 2 time points were observed among genes associated with cellular stress and altered metabolism. Since this manuscript focuses on the immune aspects of oncolytic therapy, we will restrict the discussion to immunologic signatures from now on.

**Table 3:** Mouse immune genes up-regulated in regressing GI-101A tumors at day 21 (F test p2-value < 0.001)

Gene LLID #	Symbol	Name	HT-29 Control	HT-29 GLV-1h68	GI-101A Control	GI-101A GLV-1h68

**Interleukins**						

16068	*Il18bp*	*interleukin 18 binding protein*	0.58	0.98	1.00	5.70

16173	*Il18*	*Il18 – interleukin 18*	1.24	1.25	1.00	4.10


**Chemokines**						

20296	**Ccl2**	**Ccl2/MCP-1 (human nomenclature)**	1.65	2.12	1.00	10.84

20292	**Ccl11**	**Ccl11/Eotaxin**	0.64	1.10	1.00	9.47

20307	**Ccl8**	**Ccl8/MCP-2**	1.51	3.30	1.00	7.16

	*Cxcl11*	*CXCL11/-ITAC*	0.82	1.04	1.00	5.69

17329	**Ccl12**	**Ccl12/MCP-5**	1.22	1.44	1.00	4.97

20306	*Ccl7*	*Ccl7/MCP-3.MARC*	0.72	0.90	1.00	4.71

	**Cxcl10**	**Cxcl10/IP-10**	0.83	0.84	1.00	4.08

17329	*Cxcl9*	*Cxcl9/Mig*	0.72	0.72	1.00	3.80

20308	*Ccl9*	*Ccl9/MRP-2/CCF18/MIP-1g*	1.06	1.08	1.00	3.12

20315	*Cxcl12*	*Cxcl12/SDF-1/PBSF*	0.75	0.84	1.00	3.07

20304	*Ccl5*	*Ccl5/RANTES*	0.66	0.97	1.00	2.56


**ISGs**						

16145	*Igtp*	*interferon gamma induced GTPase*	0.50	1.13	1.00	10.11

76933	*Ifi27*	*interferon, alpha-inducible protein 27*	0.87	1.14	1.00	10.04

231655	**Oasl1**	**2'-5' oligoadenylate synthetase-like 1**	0.66	0.73	1.00	6.93

17857	*Mx1*	*myxovirus (influenza virus) resistance 1*	0.77	0.89	1.00	6.41

16145	*Igtp*	*interferon gamma induced GTPase*	0.89	0.80	1.00	5.62

	*Ifi204*	*interferon activated gene 204*	0.71	1.06	1.00	5.44

246730	**Oas1a**	**2'-5' oligoadenylate synthetase 1A**	0.64	1.16	1.00	5.38

	*Iigp2*	*interferon inducible GTPase 2*	0.63	0.83	1.00	5.25

26388	**Ifi202b**	**interferon activated gene 202B**	0.54	1.17	1.00	4.61

	*Ifi47*	*interferon gamma inducible protein 47*	0.54	0.67	1.00	4.56

15957	*Ifit1*	*interferon-induced protein with tetratricopeptide repeats 1*	1.06	0.94	1.00	4.15

20847	**Stat2**	**signal transducer and activator of transcription 2**	0.81	0.85	1.00	4.06

20846	*Stat1*	*signal transducer and activator of transcription 1*	0.74	0.68	1.00	4.02

16362	*Irf1*	*interferon regulatory factor 1*	0.91	1.21	1.00	3.78

65972	**Ifi30**	**interferon gamma inducible protein 30**	0.64	0.70	1.00	3.67

246728	**Oas2**	**2'-5' oligoadenylate synthetase 2**	1.08	1.10	1.00	3.29


**Other**						

17067	*Ly6c*	*lymphocyte antigen 6 complex, locus C*	0.90	0.99	1.00	5.77

17067	*Ly6c*	*Lymphocyte antigen 6 complex, locus C*	0.95	1.12	1.00	5.27

17071	*Ly6f*	*lymphocyte antigen 6 complex, locus F*	1.01	1.08	1.00	5.01

20715	**Serpina3g**	**serine (or cysteine) peptidase inhibitor, clade A, member 3G**	0.88	0.68	1.00	4.77

18636	**Cfp**	**complement factor properdin**	0.75	0.86	1.00	4.62

15331	**Hmgn2**	**high mobility group nucleosomal binding domain 2**	1.05	1.05	1.00	4.30

13032	**Ctsc**	**cathepsin C**	0.60	0.60	1.00	4.24

64685	**Nmi**	**N-myc (and STAT) interactor**	1.04	1.11	1.00	4.12

20343	*Sell*	*selectin, lymphocyte*	0.95	0.75	1.00	3.91

12370	**Casp8**	**caspase 8**	0.82	0.51	1.00	3.74

16423	***Cd47***	***CD47 antigen (Rh-related antigen, integrin-associated signal transducer)***	0.89	0.83	1.00	3.72

14962	**Cfb**	**complement factor B**	0.77	0.89	1.00	3.58

	**Pla2g7**	**phospholipase A2, group VII (platelet-activating factor acetylhydrolase, plasma)**	0.66	0.53	1.00	3.55

13025	*Ctla2b*	*Cytotoxic T lymphocyte-associated protein 2 beta*	0.64	1.03	1.00	3.40

18595	**Pdgfra**	**platelet derived growth factor receptor, alpha polypeptide**	0.98	1.99	1.00	3.34

12267	**C3ar1**	**complement component 3a receptor 1**	0.72	0.84	1.00	3.34

16653	**Kras**	**v-Ki-ras2 Kirsten rat sarcoma viral oncogene homolog**	2.15	1.36	1.00	3.22

64138	**Ctsz**	**cathepsin Z**	0.50	0.43	1.00	3.11

* In italic transcripts common to day 21 and 42; in bold those unique to each category

**Table 4:** Mouse immune genes up-regulated in regressing GI-101A tumors at day 42 (F test p2-value < 0.001)

Gene LLID #	Symbol	Name	HT-29 Control	HT-29 GLV-1h68	GI-101A Control	GI-101A GLV-1h68	GI-101A GLV-1h68 (day 21)

**Interleukins**							

16068	*Il18bp*	*interleukin 18 binding protein*	1.31	2.18	1.00	13.28	5.70

16173	*Il18*	*interleukin 18*	1.12	1.40	1.00	10.89	4.10

16168	**Il15**	**interleukin 15**	1.02	1.51	1.00	5.20	2.86

16154	**Il10ra**	**interleukin 10 receptor alpha**	0.74	0.90	1.00	3.51	2.08


**Chemokines**							

	*Cxcl11*	*Cxcl11/I-TAC*	0.92	1.75	1.00	13.57	5.69

17329	*Cxcl9*	*Cxcl9/Mig*	1.01	1.07	1.00	11.74	3.80

20304	*Ccl5*	*Ccl5/RANTES*	1.00	2.69	1.00	13.33	3.23

20308	*Ccl9*	*Ccl9/MRP-2/CCF18/MIP-1g*	1.56	3.14	1.00	12.03	6.60

20304	*Ccl5*	*Ccl5/RANTES*	1.11	2.57	1.00	9.81	2.56

20306	*Ccl7*	*Ccl7/MARC*	0.84	1.18	1.00	5.86	4.71

20315	*Cxcl12*	*Cxcl12/SDF-1/PBSF*	0.41	0.58	1.00	5.23	3.07

20301	**Ccl27**	**Ccl27/ALP/CTACK/ILC/Eskine**	1.86	1.91	1.00	5.17	1.66

20308	*Ccl9*	*Ccl9/MRP-2/CCF18/MIP-1g*	1.26	1.42	1.00	4.04	3.12


**ISGs**							

16145	*Igtp*	*interferon gamma induced GTPase*	1.15	3.31	1.00	48.21	10.11

76933	*Ifi27*	*interferon, alpha-inducible protein 27*	0.76	0.91	1.00	12.84	10.04

	*Ifi47*	*interferon gamma inducible protein 47*	0.66	0.94	1.00	11.09	4.56

	*Iigp2*	*interferon inducible GTPase 2*	0.64	1.41	1.00	10.05	5.25

16145	*Igtp*	*interferon gamma induced GTPase*	0.70	1.28	1.00	9.70	5.62

17857	*Mx1*	*myxovirus (influenza virus) resistance 1*	0.62	1.46	1.00	9.46	6.41

	*Ifi204*	*interferon activated gene 204*	0.86	1.77	1.00	8.94	5.44

16362	*Irf1*	*interferon regulatory factor 1*	0.59	0.98	1.00	7.28	3.78

20846	*Stat1*	*signal transducer and activator of transcription 1*	0.57	0.81	1.00	6.84	4.02

20846	*Stat1*	*signal transducer and activator of transcription 1*	0.66	0.97	1.00	6.17	2.79

15957	*Ifit1*	*interferon-induced protein with tetratricopeptide repeats 1*	0.79	1.03	1.00	5.77	4.15

60440	**Iigp1**	**interferon inducible GTPase 1**	0.77	1.00	1.00	4.19	3.00

15976	**Ifnar2**	**Interferon (alpha and beta) receptor 2**	1.10	1.60	1.00	3.73	2.19

	**Irf5**	**interferon regulatory factor 5**	0.53	0.70	1.00	3.47	2.02


**Others**							

17071	*Ly6f*	*Lymphocyte antigen 6 complex, locus F*	0.56	0.73	1.00	8.56	2.68

11629	**Aif1**	**allograft inflammatory factor 1**	0.90	1.33	1.00	8.46	2.62

17067	*Ly6c*	*Lymphocyte antigen 6 complex, locus C*	1.24	1.22	1.00	7.35	5.27

17067	*Ly6c*	*lymphocyte antigen 6 complex, locus C*	0.99	1.12	1.00	6.03	5.77

17071	*Ly6f*	*lymphocyte antigen 6 complex, locus F*	0.80	0.89	1.00	5.66	5.01

20343	*Sell*	*selectin, lymphocyte*	0.61	0.79	1.00	5.03	3.91

76281	**Tax1bp1**	**Tax1 (human T-cell leukemia virus type I) binding protein 1**	1.01	1.51	1.00	5.02	1.98

230233	**Ikbkap**	**inhibitor of kappa light polypeptide enhancer in B-cells**	0.81	0.93	1.00	4.55	1.43

110454	**Ly6a**	**lymphocyte antigen 6 complex, locus A**	0.96	0.98	1.00	4.13	2.42

13025	*Ctla2b*	*Cytotoxic T lymphocyte-associated protein 2 beta*	0.68	1.20	1.00	4.12	3.40

71966	**Nkiras2**	**NFKB inhibitor interacting Ras-like protein 2**	1.07	1.27	1.00	3.80	1.93

17087	**Ly96**	**lymphocyte antigen 96**	1.13	1.43	1.00	3.77	1.44

17069	**Ly6e**	**Lymphocyte antigen 6 complex, locus E**	0.77	1.15	1.00	3.28	1.87

* In italic transcripts common to day 21 and 42; in bold those unique to each category

In general, immunologic differences between the early (day 21 from VACV injection) and the later (day 42) time points were quantitative rather than qualitative, Therefore, although some transcripts from VACV infections were significantly up regulated at day 21 a similar trend could be observed at day 42 though it did not reach the same level of statistical significance (F-test p-value < 0.001). Additionally, in the majority of cases, the host's transcription was enhanced at the later time point when tumor growth reached a plateau and the rejection process was presumed to start.

Among interleukins, IL-18 and the IL-18 binding protein played a prominent role early in the course of infection, while later IL-15 became increasingly up regulated (Table 3 and 4). CCR2, CCR3 and CCR5 ligand chemokines played a predominant role at day 21 while CXCR3 and CXCR4 ligand chemokines up-regulation became more prominent later. Among the CXCL chemokines, CXCL-12/SDF-1 was previously associated with the rejection of metastatic melanoma during IL-2 therapy [31] and together with CXCL-9 through -11 chemokines in association with the rejection of basal cell carcinomas (BCCs) treated with TLR7 agonists [32]. ISGs and other genes associated with the IFN signaling were among the most up-regulated at either time point studied; these included IFN-γ induced GTPase, whose expression was increased 48-fold at day 42 in GI-101A tumors from GLV-1h68-infected animals compared to controls. IPA suggested that the majority of up-regulated genes reflected predominantly IFN-γ stimulation, a phenomenon we have observed in BCCs regression upon treatment with TLR-7 agonists [32] and, more generally, in association with TSD [2]; among them, STAT-1 and IRF-1 were previously described in association with TSD [2,31,32] and play a central role in the signaling of IFN-γ and other pro-inflammatory cytokines such as IL-2 and IL-15 [33].

**Figure 4:**
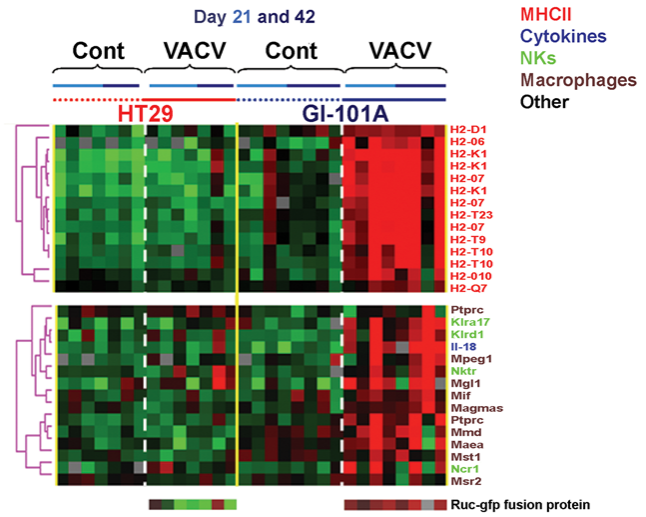
**Mouse immune gene signatures**. Self-organizing heat map based on genes selected according to macrophage (brown), natural killer cell (light green), cytokine (blue) or major histocompatiblity class II (red) annotations among those up-regulated in GI-101A xenografts excised from VACV-infected animals (Student *t *test p_2_-value < 0.001). Genes presented in Figure 6 as representative of the ICR were omitted here to avoid redundancy.

**Figure 5:**
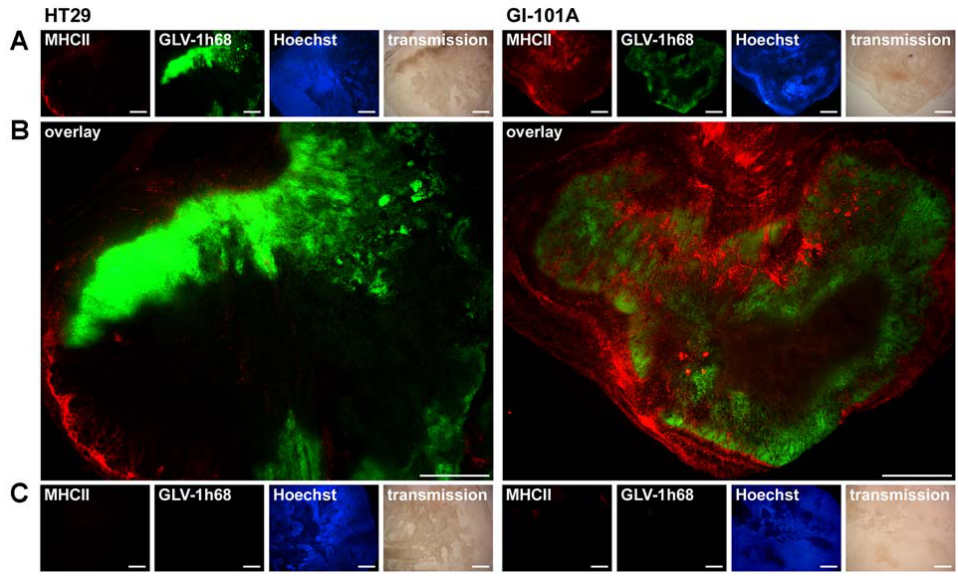
**Immunohistochemistry staining of MHC class II positive cells**. Scale bars are equal to 1 mm and 10× magnification was applied. (**A**) 42 days after GLV-1h68 administration HT-29 (left) and GI-101A (right) xenografts were excised, sectioned and labeled for MHCII and vital DNA (Hoechst). In addition, GFP signals from VACV infected cells and transmission images are shown. (**B**) Overlay of MHCII and GFP signals in HT-29 tumors (left) and GI-101A tumors (right). (**C**) Uninfected HT-29 (left) and GI-101A (right) xenografts were excised at day 42 and treated identical to their infected counterparts. Tissue sections were stained for MHCII and vital DNA (Hoechst). As expected, no Virus-derived GFP signal could be detected.

Macrophage presence/function also played an important role (Figure 4) and was associated with over-expression of major histocompatiblity class II genes supporting the presence of activated macrophages in infected GI-101A xenografts. Furthermore, this prominent and specific infiltration could be substantiated by immunohistochemical analyses that demonstrated a strong peri- and intra-tumoral infiltration of MHC class II-expressing host's cells surrounding virally-infected cancer cells (Figure 5).

**Figure 6:**
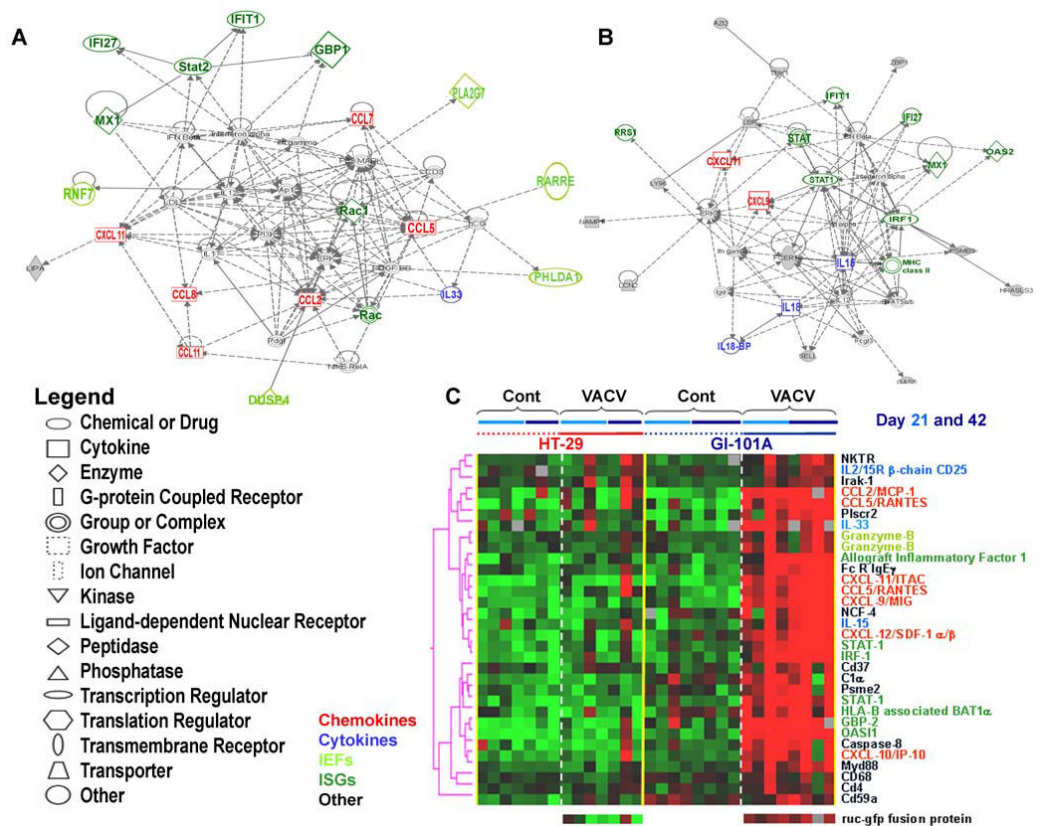
**Mouse immune genes associated with the Immunologic Constant of Rejection Hypothesis**. IPA self-organizing network based on genes with immune annotations whose expression was significantly up-regulated in VACV-infected GI-101A xenografts at day (**A**) 21 and (**B**) 42 from VACV-infection. (**C**) Self-organizing heat map based on genes associated with the ICR hypothesis. The genes were arbitrarily selected a priori based on previous studies as summarized in [2] and are displayed based on their expression in the current study without further selection.

Although there was only partial overlap between genes up-regulated at day 21 and 42, most overlap was due to genes related to immune function. Applying a stringent Student *t *test (p_2_-value < 0.001) comparing infected to non-infected GI-101A xenografts at the 2 time points, similar results were observed; although less genes were significantly up-regulated at day 21 (compared to the F test) a good proportion overlapped with day 42 and those overlapping genes were exclusively related to immune function (Table 2). We then re-directed genes with immune function significantly up-regulated in infected GI-101A xenografts into self-organizing biological pathways using IPA; this analysis identified those genes most tightly associated with the immunological network leading to TSD during rejection of GI-101A xenografts; while at day 21 (Figure 6A) CCL chemokines and STAT-2 played a central role, at day 42, IL-15, STAT-1 and IRF-1 played a central role (Figure 6B). Interestingly, IL-18, which was identified as playing a central role in this immune-deficient mouse model; was not previously observed as a component of the ICR in immune competent human tissues affected by immune-pathology. Finally, as expected no genes associated with B or T cell signaling or function in the grafts were significantly up-regulated at this phase of the immune-response against infected GI-101A xenografts, in accordance with the biology of the host's model system. This data suggest that at least in this model, adaptive immunity is not necessary for TSD.

Based on GeneOntology annotations, we then compiled a database of genes associated to TSD in various immune pathologies in accordance to the ICR hypothesis [2,34]. These genes have been described as highly associated with TSD in the context of acute allograft rejection, pathogen clearance during acute infection and tumor rejection during immunotherapy [2]. We displayed these *a priori *and arbitrarily selected genes as a self organizing heat map based on the data from the present study (Figure 6C). All were specifically expressed in infected responding tumors compared with non-infected GI-101A and the infected or non-infected HT-29 xenografts. This display represents a prospective validation of a universal mechanism leading to TSD in mice as well as in humans.

## Discussion

Immune-mediated TSD is the ultimate manifestation of the effector phase of the immune response and, as we recently argued, may follow a common final pathway independent of the pathological circumstances leading to its occurrence [2,34]. Thus, although the mechanisms originating acute allograft rejection, clearance of pathogens during acute infection, flares of autoimmunity or cancer regression may be different, in the end, they all converge into a cascade of immunologic steps capable of turning a chronic and lingering inflammatory process into an acute and destructive one. We argued that, among the 4 axioms upon which the ICR is founded, adaptive immune responses are neither necessary nor sufficient to induce tissue-specific rejection but rather start a tissue-specific reaction in cases in which such specificity is not determined by other factors. Indeed, others have shown that tumor rejection can be determined by innate immune mechanisms [3] and adaptive T cell responses play a role as helpers to stimulate more powerful innate immune effector mechanisms [20]. Thus, in conditions in which a switch from a chronic to an acute inflammatory process can be induced by other factors like the immune-stimulation induced by the presence of a virus in the target tissue, adaptive immune responses may not be necessary and immune-mediate rejection can occur without the assistance of T or B cells.

This hypothesis is suggested by some human observations. The rejection of skin cancers by the local application of TLR-7 agonists occurs without direct evidence of adaptive immune responses [32,35-37]. Also renal cell carcinomas are as sensitive to systemic administration of IL-2 as metastatic melanoma yet, while in the latter adaptive immune responses are easily demonstrable, in the former, they have been quite elusive, and most likely of secondary significance [38,39].

Xenografts growing in primarily T cell-depleted and secondarily B cell-deficient animals provide the best evidence that in the absence of non-self discrimination, allograft rejection does not occur. In this model, although xenografts by themselves do not provide sufficient pro-inflammatory signals to induce acute inflammation, the presence of viral replication provides the "tissue-specific trigger" that activates the immune response. According to our hypothesis, the ICR is activated when chronic inflammation is switched into an acute one. A critical step in this process is the expression of IFN-γ dependent pathways probably by activated mononuclear cells; this is clearly demonstrable in most cases in which TSD has been studied in humans by the requirement for the expression of IRF-1 [2,31,40,41]; a transcription factor closely related to IFN-γ signaling. IFN-α and IFN-γ regulate directly or indirectly the production of CXCR3, CXCR4 and CCR5 ligands among which the CXCL-9 through -11 chemokines (Mig, IP-10/Crg-2 and ITAC), CCL5 (RANTES) and CXCL12 (SDF-1) appear to play a prominent role [42]. Indeed, this expression pattern has been consistently observed in most cases in which TSD was studied in the involved tissue by transcriptional profiling [2,34] including animal rejection models [20]. This study provides experimental evidence that such signatures are associated with TSD and, potentially, immune-mediate rejection independent of the presence of adaptive immunity. Moreover, this model provides evidence that non-self discrimination plays at best a partial role in a host that cannot eliminate xenografts unless appropriate danger signals are provided by a pathogen [43-46]. Contrary to acute allograft rejection occurring in humans [47], no B lymphocyte signatures (CD20, immunoglobulin genes) could be observed clearly demonstrating that reconstitution of a potential B cell response could not have been responsible for the inflammatory switch and the production of CXCL and CCL chemokines [48]. Furthermore, contrary to a similar rejection model that we presently analyzed in a syngeneic mouse system [49], no involvement of T or B cell signatures participated in the rejection of GI-101A xenografts. Furthermore, contrary to the syngeneic model of HER-2/*neu*-expressing mammary tumor rejection [49] where clear up-regulation of type I and type II IFNs could be documented, in this model ISG expression was not directly accompanied with the over expression of IFN-α, IFN-β or IFN-γ suggesting that, as recently demonstrated in a cytomegalovirus model [50], stimulation of interferon response genes could occur independently of de novo synthesis of IFNs through a direct interaction of viral proteins with cellular transcription factors.

Although most differences in the transcriptional pattern of human cancer cells were associated with arrested or dampened metabolism (Figure 3D) a handful of genes were up-regulated specifically in infected GI-101A xenografts. Among those, only IRF-7 and STAT-3 could be proven to be specifically expressed by human cells (Figure 4B). The expression of IRF-7 is not surprising considering the presence of replicating VACV in those cells compared with control xenografts from non-infected animals [18], while the expression of STAT-3 in the absence of over-expression of STAT-1 contrasts with the analysis of host's transcripts in which STAT-1 over-expression dominated (Table 3). As IRF-7 and STAT-3 were also expressed by host cells, it could be hypothesized that transfer of VACV from cancer cells to host cells infiltrating the xenografts reproduced patterns observed in human cancer cells, while host's immune cells followed the classical up-regulation of pro-inflammatory pathways through STAT-1, IRF-1 signaling [18,42,51].

The over-expression of IL-18 and the IL-18-binding protein in this model is of particular interest. IL-18, originally called IFN-γ inducing factor, has not been previously observed by us or others as consistently associated with TSD [2]. It is possible, that IL-18 over expression is a specific causative mechanism in this model as VACV particles have been described as inducers of this cytokine by direct monocyte activation through TLR signaling [52-54] a finding that needs to be corroborated by future studies. Contrary to IL-18, IL-15 is the most consistently observed cytokine in association with TSD [2]. Generally, we have observed this in association with the expression of IL-2 and, it is of interest that, in this T cell-depleted model only this monocyte produced cytokine is present. IL-15 is critical not only in expansion of memory CD8+ T cells in mice but also to maintain cytotoxic T cell effector functions. In fact, VACV clearance is delayed in IL-15 -/- mice due to a rapid loss of cytolytic function [55] most likely by natural killer cells [56]. Thus, the role that IL-15 may play in this immune-deficient model will need to be further investigated.

In practical terms, it would be important to understand why some tumors could be eliminated through viral oncolysis and/or a secondary immune rejection, while others are resistant. It appears that the degree of viral replication *in vivo *is a key determinant; however, it remains unclear the weight that direct viral oncolysis plays compared to immune-mediated rejection in this model. It appears that transcriptional changes associated with viral replication precede tumor destruction by a substantial amount of time and they are paralleled by the activation of immune signatures in the host, long before tissue destruction occurs. For instance, viral replication was quite active at day 21 in GI-101A xenografts; at the same time significant shut down of cancer cell metabolism (Figure 3C, D) and simultaneous activation of immune functions could be observed at that early time point (Table 2). Yet, tumors continued their growth at least till day 42 when their growth started to plateau. HT-29 allowed VACV replication *in vitro *similarly to GI-101A, but *in vivo *viral replication was substantially reduced in most though not all HT-29 xenografts (Figure 2C). This suggests that although the baseline biological phenotype of individual cell lines can influence viral replication, *in vivo *other factors may interfere with viral replication, and need to be further studied. This is suggested by the significant yet imperfect correlation between *in vitro *and *in vivo *replication data and, most importantly, by the individual variation among xenografts originated from the same cell line that can be permissive or non-permissive to viral replication *in vivo *(HT-29 example in Figure 2C). To clarify such subtleties, it will be necessary to investigate a larger panel of cell lines, and assess the growth patterns of individual xenografts. This could be achieved by the utilization of non-invasive strategies such as fine needle aspirations that allow direct linkage of the experimental results obtained by transcriptional profiling to the natural or therapy-induced history of each individual xenograft left in place [31,57,58].

## Conclusion

Although xenograft infection by oncolytic VACV offers a promising therapy of established cancer, it needs to be taken into account that the presence of adaptive immunity might change what is expected, perhaps inducing suppressive T cell responses that could abrogate the therapeutic effect of the virus in natural conditions.

The rejection of GI-101A tumors seems to be mediated by infiltrating leukocytes; thus, cancer cells not greatly affected by the viral infection *in vitro *may show resistance to *in vivo *oncolytic therapy. Future studies utilizing a broader panel of cell lines will be necessary to evaluate whether a correlation exists between *in vitro *replication pattern and *in vivo *regression following VACV infection. Alternatively, other factors related to the host response within the tumor microenvironment besides the in vitro permissiveness of cell lines may affect their in vivo permissiveness to VACV and/or their pattern of growth. Indeed, the nature of the tumor microenvironment might predict the success of the VACV therapy even though the treatment outcome seems to be mainly correlated with the ability of the infected tumor cells to provide the "tissue-specific signal" to activate the immune response and attract specific leukocytes.

In summary, this study provides the first prospective validation of a universal mechanism associated with TSD. This information may lead to the identification of principles that could refine the treatment of cancer and chronic infection by immune stimulation or autoimmunity and allograft rejection through immune regulation.

## Methods

### Cell line culture

All cell lines except when noted were purchased from American Type Culture Collection (Manassas). GI-101A cells were kindly provided by Dr. A. Aller, Rumbaugh-Goodwin Institute for Cancer Research, Inc., Plantation, Florida whereas the 3 melanoma cell lines from distinct cutaneous metastases were obtained from patient 888 as previously described [24]. MDA MB-231, PANC-1, CV-1 and PC-3 cells were cultured in Dulbecco's modified Eagle's medium (DMEM) supplemented with 10% fetal bovine serum (FBS) and 1% antibiotic-antimycotic solution (AA) (100 U/ml penicillin G, 250 ng/ml amphotericin B, 100 μg/ml streptomycin). MIA PaCa-2 cells have been cultured under similar conditions in DMEM media but supplemented with 12.5% FBS and 2 mM L-glutamine. SiHa and DU-145 cells were grown in Eagle's minimal essential medium (EMEM) which was enhanced with 10% FBS, 1% non-essential amino acids (NEAA), 1 mM sodium pyruvate and 1% AA.

All other cells were cultured in Roswell Park Memorial Institute medium (RPMI) supplemented with the following compounds: A-549 and HT-29 cells (10% FBS and 1% AA); GI-101A cells (20% FBS, 4.5 g/L glucose, 10 mM HEPES, 1 mM sodium pyruvate, 1% AA and 4 ng/ml β-estradiol/5 ng/ml progesterone); NCI-H1299 (10% FBS, 4.5 g/L glucose, 10 mM HEPES, 1 mM sodium pyruvate, 1% AA). OVCAR-3 culture media was prepared similarly to GI-101A media but supplemented with 2.3 g/L glucose instead and additional human Insulin; and 888-MEL, 1858-MEL and 1936-MEL cells (10% FBS, 1 mM HEPES, 1 mM Ciprofloxacin and L-glutamine/penicillin/streptomycin). All cell cultures were carried out at 37°C under 5% CO_2_.

### Viral construct

The construction of the mutant GLV-1h68 virus was described previously [13]. Briefly, 3 expression cassettes (encoding for *Renilla *luciferase-*Aequorea *GFP fusion protein, β-galactosidase and β-glucoronidase) were recombined into the F14.5L, J2R and A56R loci, respectively, of the LIVP strain viral genome.

### *In vitro *viral replication assay

All cells were seeded in 6-well plates and infected with GLV-1h68 at the multiplicity of infection of 0.01 as we have previously described [13]. The infected cell cultures were harvested in triplicate up to 72 hours post infection (hpi). Viral titers were determined by plaque assays on CV-1 cell monolayers and expressed as pfu/10^6 ^cells.

### Virus titration of tumor tissue

GLV-1h68 infected tumors were removed at day 7, 21 and 42, weighed and homogenized in DPBS containing proteinase inhibitor cocktail using MagNALyser (Roche Diagnostics) at a speed of 6500 for 30 s. After three freeze and thaw cycles to release the viral particles, the samples were sonicated and supernatants were collected by centrifugation at 1000 g for 5 min. Viral titers were determined in duplicates by standard plaque assays using CV-1 cells.

### Animal models

All mice were cared for and maintained in accordance with animal welfare regulations under an approved protocol by the Institutional Animal Care and Use Committee of LAB Research International Inc. (San Diego Science Center, San Diego, CA). Six to 8 weeks old nude mice (NCI:Hsd:Athymic Nude-*Foxn1*^nu^, Harlan) were inoculated with 5 × 10^6 ^cells per mouse to obtain subcutaneous xenografts as previously described [13]. Tumor growth was measured once a week and tumor mass was reported in mm^3^. Thirty days after implantation, 5 × 10^6 ^pfu of GLV-1h68 virus in 100 μl of PBS or 100 μl of PBS alone (control) was delivered by intravenous inoculation [13]. After inoculation with GLV-1h68 the expression of green fluorescent protein within the tumors could be monitored under UV-light. Twenty-one days and 42 days post inoculation 3 or 4 animals from the treatment and the control groups were sacrificed and the tumors were excised.

### Immunohistochemistry

GI-101A xenografts from GLV-1h68-infected and non-infected mice were removed at day 42 and snap-frozen in liquid N_2_, followed by fixation in 4% paraformaldehyde/PBS pH 7.4 for 16 h at 4°C. Tissue sectioning was performed as previously described [59]. MHCII-positive cells were labeled using monoclonal rat anti-MHCII antibody (NatuTec, Frankfurt, Germany) and Cy3-conjugated donkey anti-rat secondary antibodies (Jackson ImmunoResearch, PA, USA). Hoechst 33342 (Sigma, Taufkirchen, Germany) was used to stain nuclei. The fluorescent-labeled sections were examined using the Leica MZ 16 FA Stereo-Fluorescence microscope equipped with a Leica DC500 Digital Camera (Leica, Solms, Germany). Digital images were processed with Photoshop 7.0 (Adobe Systems, CA, USA).

### Transcriptional profiling platforms

VACV-gene expression was assessed by a custom-made VACV array platform (VACGLa520445F, Affymetrix, CA) including 308 probes representing 219 genes that covered the combined genome of several VACV strains (see additional file # 2), the *Renilla *luciferase-*Aequorea *green fluorescent fusion gene specific for GLV-1h68, and 337 human or mouse "house keeping" genes (393 probes). Time course analysis evaluating the *in vivo *effects of viral replication on the permissive GI-101A human xenografts was performed using a previously described custom-made 17.5 k human cDNA array platform [29]. Human or mouse arrays covered the complete genome of each species based on 36,000 oligos each. We have previously observed that the use of species-specific cDNA arrays as well as oligo probes can distinguish the expression patterns in mixed cell populations in which human tissues (cancer cells) are infiltrated with host cells. This is because of a lack or reduced cross-hybridization between non-related species compared to closely related ones such as primate to primate comparisons [13]. Although partial cross-hybridization may occur this can be detected and eliminated by applying an appropriate intensity signal cutoff. Since cDNA arrays contain probes of relatively large size (600 to 2,000 bases), to increase the specificity of the hybridization, we tested the same material on custom-made 36 kb oligo array platforms constituted of 70-base-length oligo-probes as well as cDNA probes using identical statistical parameters. With few exceptions (discussed in the results section) results were concordant between platforms and will be presented, thereof, in either format while comprehensive data are accessible through GEO.

### Total RNA isolation and amplification

Total RNA (tRNA) from excised tumors was isolated after homogenization using Trizol reagent according to the manufacturer's instructions. tRNA from tissue cultures was isolated with the Qiagen RNeasy Mini kit and the quality of obtained tRNA was tested with the Agilent Bioanalyzer 2000 (Agilent Technologies). For expression studies based on cDNA and oligo array techniques, tRNA was amplified into antisense RNA (aRNA) as previously described [60,61].

Mouse reference RNA was prepared by homogenization and pooling of selected mouse tissues (lung, heart, muscle, kidneys, liver and spleen) from 3 female C57Bl/6 mice. Reference for human arrays was obtained by pooling PBMCs from 4 normal donors. Both, human and mouse reference tRNA was amplified into antisense RNA in large amounts [60,61]. Five μg tRNA of selected tumor and cell samples were amplified according to the Affymetrix manual using the GeneChip^® ^One-Cycle Target Labeling and Control kit.

### Microarray performance and statistical analysis

Array quality was documented as previously described [29]. For 36 k whole genome mouse and human array performances both reference and test aRNA were directly labeled using ULS aRNA Fluorescent Labeling kit (Kreatech) with Cy3 for reference and Cy5 for test samples and co-hybridized to the slides [49]. 17 k human cDNA arrays were carried out as described according to our standard method for labeling and array hybridization [62]. A customized VACV-GLV-1h68 Affymetrix expression array was specifically prepared for this study. Amplified RNA from tumor or cell samples was handled according to the manufacture's instructions for eukaryotic sample processing and hybridized to the arrays. After a 16 h incubation in the hybridization oven at 45°C, the arrays were washed and stained in the Fluidics station using the GeneChip^® ^Hybridization, Wash, and Stain Kit.

The data was uploaded to the mAdb databank  and further analyzed using BRBArrayTools developed by the Biometric Research Branch, National Cancer Institute [63] and Cluster and TreeView software [64]. Multiple dimensional scaling was performed on the BRB-array tool.

Data retrieved from the Affymetrix platform was normalized using median over entire array as reference because of single color labeling technology. For all array type's unsupervised analysis was used for class confirmation using the Stanford Cluster program (80% gene presence across all experiments and at least 3-fold ratio change) and Treeview program for visualization. Gene ratios were average corrected across experimental samples and displayed according to uncentered correlation algorithm. Class comparison was performed using parametric unpaired Student's t test or 3-way ANOVA to identify differentially expressed genes among GLV-1h68 infected and uninfected tumors or cells at various time points using different significance cutoff levels as demanded by the statistical power of each test. Subsequent filtering (80% gene presence across all experiments and at least 3-fold ratio change) narrowed down the number of genes that were expressed differentially between experimental groups.

Statistical significance and adjustments for multiple test comparisons were based on univariate and multivariate permutation test as previously described.

No quantitative polymerase chain reaction-based (q-PCR) validation of the gene sets identified in this study was performed since we have previously extensively shown that the present method for RNA amplification is robust and yields results comparable to those obtained by qPCR [29,65,66], and the primary purpose of the analysis was to evaluate general patterns of expression rather than identifying and characterize single gene expression levels.

Gene function interpretation was based on GeneOntology software while pathway analysis was based on Ingenuity Pathways Analysis software.

### Sequence analysis

To determine species origin of selected genes we designed primers flanking array probe positions within coding regions (Additional file 2). Gene transcript sequences were obtained from Ensembl database. To rule out any cross hybridizations we did extensive BLAST search of the designed primer sequences.

Specific primers were used to reverse transcribe 500 ng tRNA from excised tumors and amplify the messages subsequently. The resulting PCR products were analyzed with the Agilent Bioanalyzer to proof their length and presence. All amplicons have been cleaned up with ExoSAP-IT^® ^(United States Biomedical/Affymetrix, Cleveland, OH, USA) and transferred to the sequencing reactions performed with BigDye^® ^. Before loading into the 48-capillary 3730 DNA Analyzer (Applied Biosystem, Foster City, CA, USA) all reactions were purified with DyeEx 2.0 Spin Kit (Qiagen, Valencia, CA, USA).

## Competing interests

this work was supported by Genelux Co.; Andrea Worschech, Nanhai Chen, Yong A Yu, Qian Zhang, Stephanie Weibel, Viktoria Raab and Aladar A Szalay have received payment or are employees of Genelux Co.

## Authors' contributions

AW performed experiments, data analysis, conceived and designed the study and wrote the paper under supervision of EW, AAS and FMM who designed the study and drafted the paper. NC, YAY, QZ helped to design the study and performed some experiments. VM, RMB, MS and DFS contributed to the interpretation of the results and reviewed the paper. ZP, SW, VR, AM and HL participated or helped to carry out some experiments. All authors read and approved the final manuscript.

## Supplementary Material

Additional file 1 **Replication ability of GLV-1h68 in multiple human cancer cell lines**. Viral titers were examined 24, 48 and 72 hpi in 13 human cancer cell lines representing highly susceptible and delayed *in vitro *replication models. Click here for file

Additional file 2 **Sequence verification of genes which have potentially cross hybridized between human and mouse arrays**. The data provided represent the sequence analysis of selected genes which have been described as up regulated in infected GI-101A xenografts based on human 17 K cDNA arrays but not based on human 36 K oligo arrays. Some of the genes were in fact expressed by the host and cross-hybridized to less specific cDNA probes on the human platform. Click here for file
