# Supplementary material for: Systemic treatment of xenografts with vaccinia virus GLV-1h68 reveals the immunologic facet of oncolytic therapy
Source: BMC Genomics. 2009 Jul 7;10:301. doi: 10.1186/1471-2164-10-301 (PMC2713268; doi:10.1186/1471-2164-10-301)

Additional file 2

In vitro replication ability of GLV-1h68 in different human cancer cells lines. Viral titers in infected cell cultures were determined in triplicates by plaque assays 24, 48 and 72 hours post infection and 2 replication patterns could be distinguished. 3 cell lines (red box) resisted replication during the first 24 hours whereas 10 cell lines (green box) allowed GLV-1h68 replication even early within the first hours.

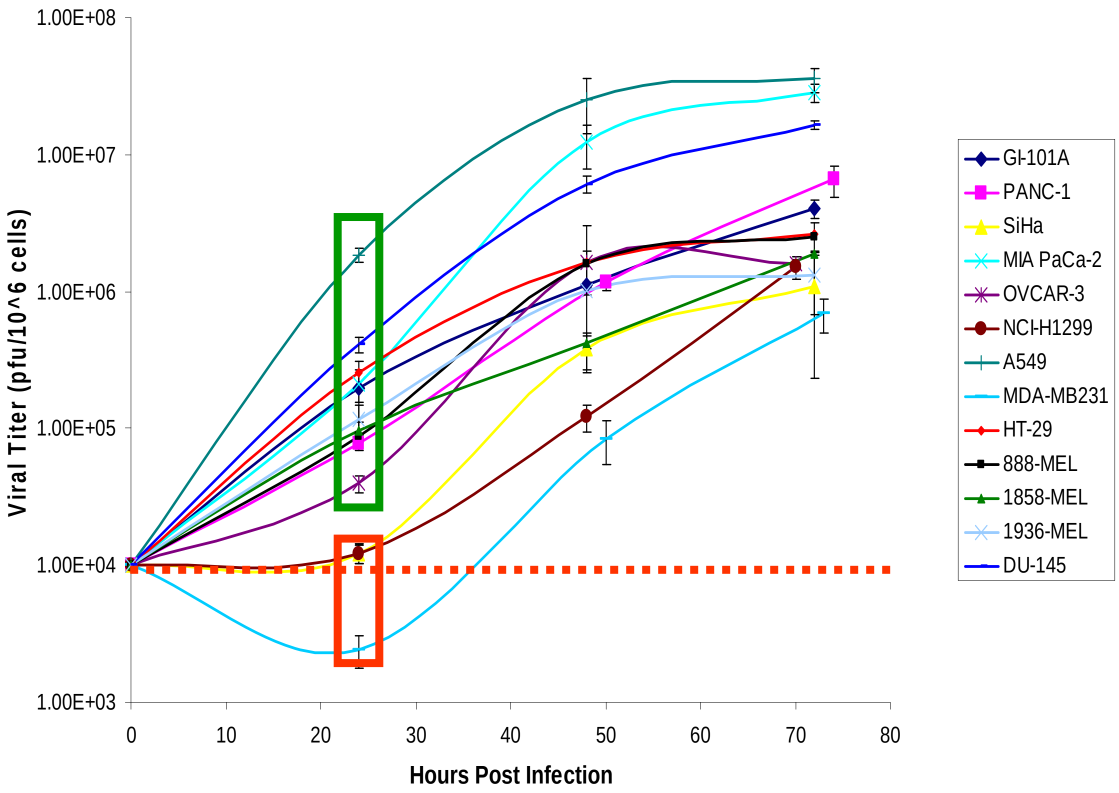

Supplement: Additional file 1 — Replication ability of GLV-1h68 in multiple human cancer cell lines. Viral titers were examined 24, 48 and 72 hpi in 13 human cancer cell lines representing highly susceptible and delayed in vitro replication models. [file 1471-2164-10-301-S1.pdf]
