# Supplementary material for: Systemic treatment of xenografts with vaccinia virus GLV-1h68 reveals the immunologic facet of oncolytic therapy
Source: BMC Genomics. 2009 Jul 7;10:301. doi: 10.1186/1471-2164-10-301 (PMC2713268; doi:10.1186/1471-2164-10-301)
Supplement: Additional file 2 — Sequence verification of genes which have potentially cross hybridized between human and mouse arrays. The data provided represent the sequence analysis of selected genes which have been described as up regulated in infected GI-101A xenografts based on human 17 K cDNA arrays but not based on human 36 K oligo arrays. Some of the genes were in fact expressed by the host and cross-hybridized to less specific cDNA probes on the human platform. [file 1471-2164-10-301-S2.pdf]

Additional file 1

Sequence verification of genes differentially expressed by GI-101A infected compared to non-infected tumors according to both human and mouse arrays. (**A**) Primer pair specificity for human and mouse genes tested on PBMC amplified RNA; bp size of the predominant band is shown at the bottom of each Agilent display in black when corresponding to the expected size and in red when discrepant. The boxes outline the corresponding species primer pair, PBMC matches. (**B**) Agilent Bio-analyzer display of amplicons from amplified RNA from three infected GI-101 tumors (2885, 2886, 2888) using the same probes and display as shown in (**A**). Red boxes outline human genes identified in the xenografts and the blue mouse genes. All amplicons were sequence verified to belong to the respective species.

Primers used for sequence analysis

	**positivestrand**
AIF1-humanfor1	GCAGAGGAGGAAGGAATGAGG
AIF1-humanrev1	GCAGGAAGAGAGGCTGGATGA
AIF1-humanfor2	TGCCCTCCAAACTGGAAGGC
AIF1-humanrev2	GCTATCTCTGAGTTGCCCTGAT
Aif1-mousefor	GCCAACTGGTCCCCCAGC
Aif1-mouserev	ATAAATGACGCTCCTAGTGGGTC
IRF7-humanfor	TGTGCCGAGTGCACCTAGAG
IRF7-humanrev	CGCGGCCGCCCAGTCTAA
Irf7-mousefor	GAGCCTCAGCAATGCTCTGC
Irf7-mouserev	AGCAAGACCGTGTTTACGAGGAA
TLR2-humanfor	TGTGCCACCGTTTCCATGGC
TLR2-humanrev	GACTTCATTCCTGGCAAGTGGAT
Tlr2-mousefor	CTGGAGAACTCTGACCCGC
Tlr2-mouserev	GAGAACAACGACGCGGCCAT
STAT3-humanfor1	ATTGACCTTGTGAAAAAGTACATCC
STAT3-humanrev1	CGTGGAACCATACACAAAGCAG
STAT3-humanfor2	CCCCATACCTGAAGACCAAGTTT
STAT3-humanrev2	CACAGCCAAACCCCAGATCAT
Stat3-mousefor	CCGTACCTGAAGACCAAGTTC
Stat3-mouserev	GGGTGATAAACTGTTATGTAAAGAG
TNF-humanfor	AGGAGGACGAACATCCAACCTT
TNF-humanrev	CACTGGGGCCTACAGCTTTGA
Tnf-mousefor	TAGAAAGGGGATTATGGCTCAGG
Tnf-mouserev	CAGATTCTTCCCTGAGGTGCAA

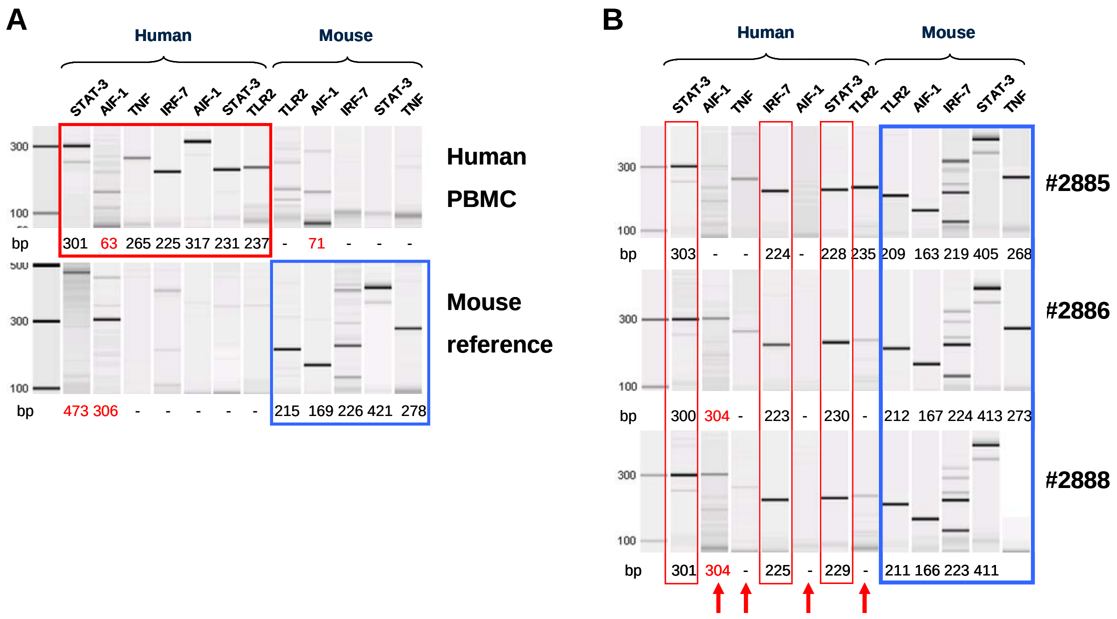

A
Human
Mouse
STAT-3 AIF-1 TNF IRF-7 AIF-1 STAT-3 TLR2 TLR2 AIF-1 IRF-7 STAT-3 TNF
300
100
bp
301 63 265 225 317 231 237 - 71 - - -
Human
PBMC
500
300
100
bp
473 306 - - - - - 215 169 226 421 278
Mouse
reference
B
Human
Mouse
STAT-3 AIF-1 TNF IRF-7 AIF-1 STAT-3 TLR2 TLR2 AIF-1 IRF-7 STAT-3 TNF
300
100
bp
303 - - 224 - 228 235 209 163 219 405 268
#2885
300
100
bp
300 304 - 223 - 230 - 212 167 224 413 273
#2886
300
100
bp
301 304 - 225 - 229 - 211 166 223 411
#2888
